# Zanubrutinib Versus Ibrutinib in Symptomatic Waldenström Macroglobulinemia: Final Analysis From the Randomized Phase III ASPEN Study

**DOI:** 10.1200/JCO.22.02830

**Published:** 2023-07-21

**Authors:** Meletios A. Dimopoulos, Stephen Opat, Shirley D'Sa, Wojciech Jurczak, Hui-Peng Lee, Gavin Cull, Roger G. Owen, Paula Marlton, Björn E. Wahlin, Ramon Garcia-Sanz, Helen McCarthy, Stephen Mulligan, Alessandra Tedeschi, Jorge J. Castillo, Jaroslaw Czyz, Carlos Fernández de Larrea, David Belada, Edward Libby, Jeffrey Matous, Marina Motta, Tanya Siddiqi, Monica Tani, Marek Trněný, Monique C. Minnema, Christian Buske, Veronique Leblond, Steven P. Treon, Judith Trotman, Wai Y. Chan, Jingjing Schneider, Heather Allewelt, Sheel Patel, Aileen Cohen, Constantine S. Tam

**Affiliations:** ^1^ National and Kapodistrian University of Athens, Athens, Greece; ^2^ Monash Health & Monash University, Clayton, VIC, Australia; ^3^ Centre for Waldenström's Macroglobulinemia & Associated Disorders, University College London Hospital Foundation Trust, London, United Kingdom; ^4^ Maria Sklodowska-Curie National Institute of Oncology, Krakow, Poland; ^5^ Flinders Medical Centre, Adelaide, SA, Australia; ^6^ Sir Charles Gairdner Hospital, University of Western Australia, Perth, WA, Australia; ^7^ St James University Hospital, Leeds, United Kingdom; ^8^ Princess Alexandra Hospital and University of Queensland, Brisbane, QLD, Australia; ^9^ Karolinska Universitetssjukhuset & Karolinska Institutet, Stockholm, Sweden; ^10^ Hospital Universitario de Salamanca, Salamanca, Spain; ^11^ Royal Bournemouth & Christchurch Hospital, Bournemouth, United Kingdom; ^12^ Royal North Shore Hospital, Sydney, NSW, Australia; ^13^ ASST Grande Ospedale Metropolitano Niguarda, Milan, Italy; ^14^ Dana-Farber Cancer Institute, Boston, MA; ^15^ Collegium Medicum in Bydgoszcz, Nicolaus Copernicus University in Toruń, Bydgoszcz, Poland; ^16^ Hospital Clínic de Barcelona, IDIBAPS, Barcelona, Spain; ^17^ FN Hradec Králové, Hradec Králové, Czechia; ^18^ Fred Hutchinson Cancer Center, Seattle, WA; ^19^ Colorado Blood Cancer Institute, Denver, CO; ^20^ AO Spedali Civili di Brescia, Lombardia, Italy; ^21^ City of Hope National Medical Center, Duarte, CA; ^22^ Ospedale Civile Santa Maria delle Croci, AUSL Ravenna, Ravenna, Italy; ^23^ Všeobecná fakultní nemocnice v Praze, Prague, Czechia; ^24^ University Medical Center Utrecht, Utrecht, the Netherlands; ^25^ Institute of Experimental Cancer Research —CCC Ulm—Universitätsklinikum Ulm, Ulm, Baden-Württemberg, Germany; ^26^ Sorbonne University, Pitié Salpêtrière Hospital, Paris, France; ^27^ Concord Repatriation General Hospital, Sydney, NSW, Australia; ^28^ BeiGene USA, Inc, San Mateo, CA; ^29^ The Alfred Hospital, Melbourne, VIC, Australia

## Abstract

The phase III ASPEN study demonstrated the comparable efficacy and improved safety of zanubrutinib versus ibrutinib in patients with Waldenström macroglobulinemia (WM). Here, we report long-term follow-up outcomes from ASPEN. The primary end point was the sum of very good partial response (VGPR) + complete response (CR) rates; secondary and exploratory end points were also reported. Cohort 1 comprised 201 patients (myeloid differentiation primary response 88–mutant WM: 102 receiving zanubrutinib; 99 receiving ibrutinib); cohort 2 comprised 28 patients (myeloid differentiation primary response 88 wild-type WM: 28 zanubrutinib; 26 efficacy evaluable). At 44.4-month median follow-up, VGPR + CR rates were 36.3% with zanubrutinib versus 25.3% with ibrutinib in cohort 1 and 30.8% with one CR in cohort 2. In patients with CXC motif chemokine receptor 4 mutation, VGPR + CR rates were 21.2% with zanubrutinib versus 10.0% with ibrutinib (cohort 1). Median progression-free survival and overall survival were not reached. Any-grade adverse events (AEs) of diarrhea (34.7% *v* 22.8%), muscle spasms (28.6% *v* 11.9%), hypertension (25.5% *v* 14.9%), atrial fibrillation/flutter (23.5% *v* 7.9%), and pneumonia (18.4% *v* 5.0%) were more common with ibrutinib versus zanubrutinib; neutropenia (20.4% *v* 34.7%) was less common with ibrutinib versus zanubrutinib (cohort 1). Zanubrutinib was associated with lower risk of AE-related treatment discontinuation. Overall, these findings confirm the long-term response quality and tolerability associated with zanubrutinib.

## Teaser

Zanubrutinib continues to demonstrate meaningful efficacy and favorable safety in patients with WM.

## INTRODUCTION

Zanubrutinib is a potent, selective next-generation covalent Bruton tyrosine kinase inhibitor approved in several countries for Waldenström macroglobulinemia (WM) in adults.^1-4^ Despite not meeting its primary end point at a median follow-up of 19.4 months in ASPEN, zanubrutinib demonstrated comparable efficacy and favorable safety compared with ibrutinib.^5^ With 2 years of additional follow-up in ASPEN, we present long-term efficacy and safety analyses.

## METHODS

The open-label, phase III ASPEN study (ClinicalTrials.gov identifier: NCT03053440) compared ibrutinib versus zanubrutinib in patients with WM. Cohort 1 included patients with mutant myeloid differentiation primary response 88 (*MYD88*^MUT^) randomly assigned 1:1 to zanubrutinib 160 mg twice daily or ibrutinib 420 mg once daily; cohort 2 included patients with wild-type *MYD88* (*MYD88*^WT^) who received zanubrutinib 160 mg twice a day.^5^ Study design, methods, and primary analysis results have been described.^5,6^ The ASPEN study was approved by the independent institutional review board or independent ethics committee at each study site and was conducted in accordance with applicable regulatory requirements, the principles of the Declaration of Helsinki, and Good Clinical Practice guidelines of the International Conference on Harmonization. All patients provided written informed consent.

## RESULTS

### Patient Disposition and Characteristics

**FIG 1.**
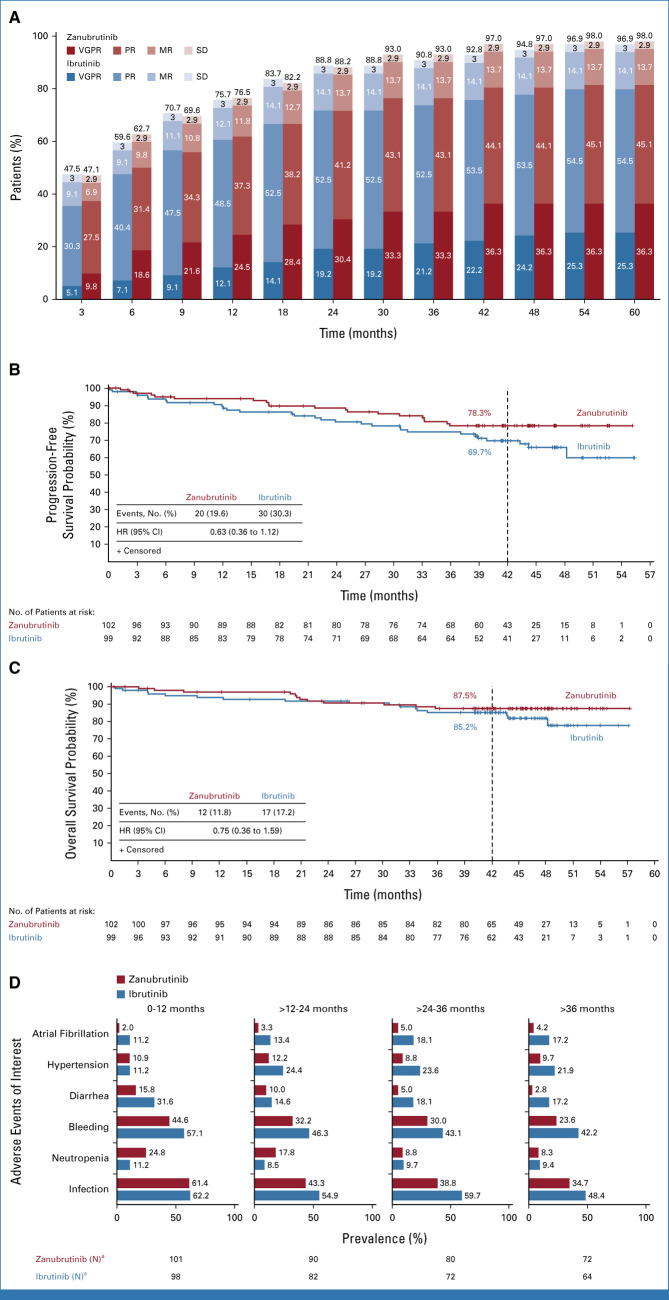
(A) Best overall response rates over time as assessed by investigator, (B) progression-free survival, and (C) overall survival in the intent-to-treat patients (99 receiving ibrutinib; 102 receiving zanubrutinib at each time point) and (D) prevalence analysis for adverse events of interest from 0 to >36 months (cohort 1). Data cutoff: October 31, 2021. ^a^N is the number of patients who are on treatment in each time interval or who discontinued treatment. The time from first dose date to the earliest date (last dose date + 30 days, initiation of new anticancer therapy, end of study, death or cutoff date) is within the time interval. The prevalence of each interval is the No. of patients with a new or ongoing event during the interval, shown as % of N. HR, hazard ratio; MR, minor response; PR, partial response; SD, stable disease; VGPR, very good partial response.

From January 2017 to July 2018, 201 patients with *MYD88*^MUT^ WM were enrolled in cohort 1 (102 receiving zanubrutinib; 99 receiving ibrutinib); 28 patients were enrolled in cohort 2 (26 *MYD88*^WT^; two unknown). More patients randomly assigned to zanubrutinib than ibrutinib were older than 75 years (33.3% *v* 22.2%, respectively; *P* = .084) and had CXC motif chemokine receptor 4 mutation (*CXCR4*^MUT^) disease (32.4% *v* 20.2%, respectively; Table 1; *P* = .073). At a median follow-up of 44.4 months (range, 0.4-57.3), 65.7% of patients on zanubrutinib and 51.5% on ibrutinib remained on treatment (cohort 1). At a median follow-up of 42.9 months (range, 2.3-53.7), 35.7% of patients remained on zanubrutinib (cohort 2; Data Supplement, Fig 1 [online only]).

**TABLE 1.**
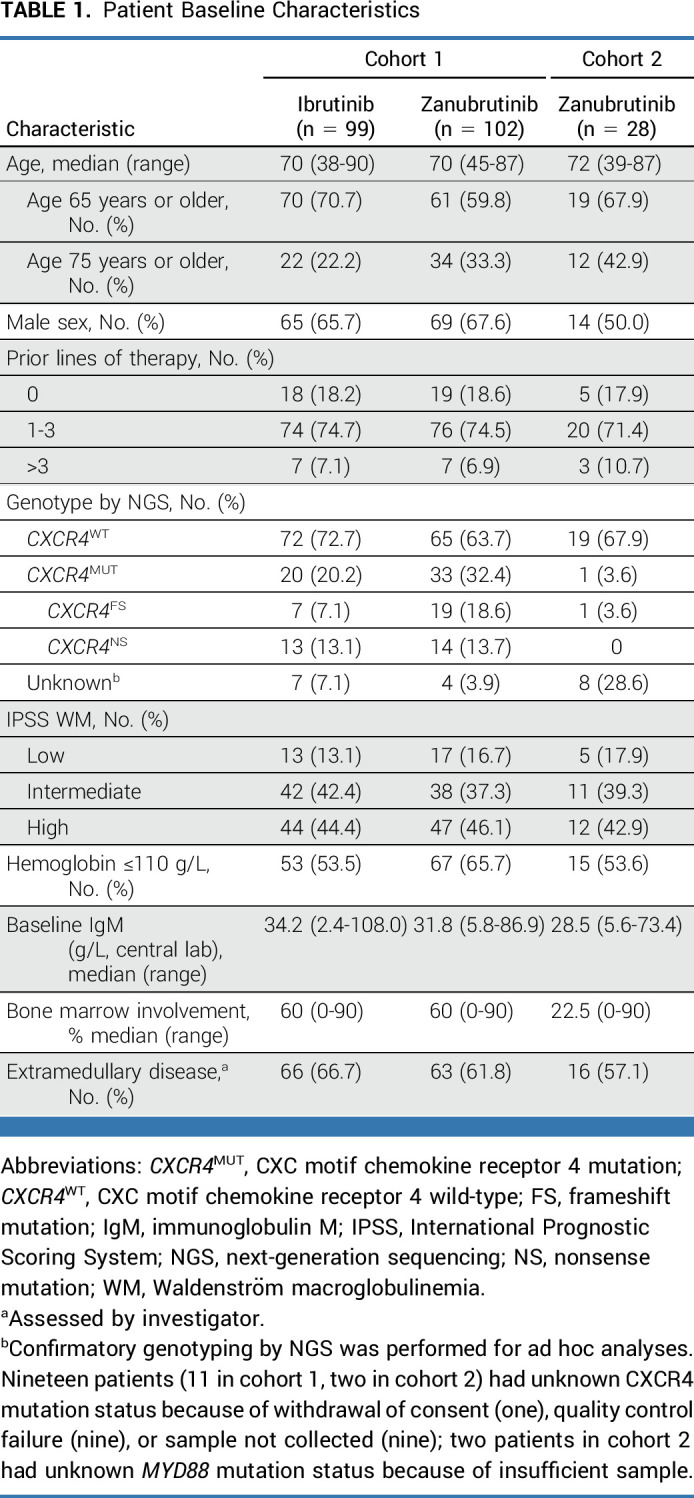
Patient Baseline Characteristics

### Efficacy

Very good partial response (VGPR) rates increased over time and were numerically higher with zanubrutinib than ibrutinib at all time points (Fig 1A). The median time to VGPR was faster for patients on zanubrutinib (6.7 months) versus ibrutinib (16.6 months); the median time to overall (minor response or better) or major (partial response or better) responses were similar between arms. Median durations of response were not reached (Table 2).

**TABLE 2.**
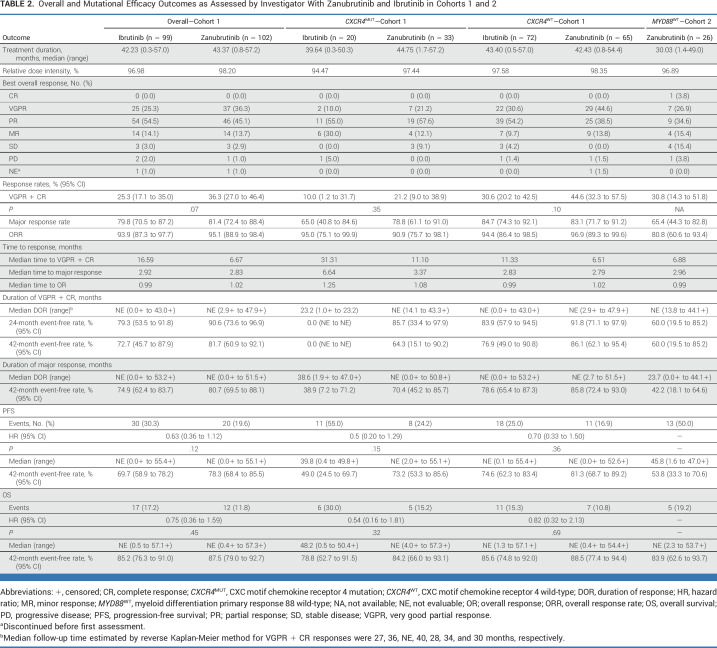
Overall and Mutational Efficacy Outcomes as Assessed by Investigator With Zanubrutinib and Ibrutinib in Cohorts 1 and 2

In patients with *CXCR4*^MUT^, higher major response rates and faster median time to response were observed with zanubrutinib versus ibrutinib (Table 2). Regardless of *CXCR4* mutational status or mutation type (nonsense *v* frameshift), VGPR + complete response (CR) rates were numerically higher for zanubrutinib versus ibrutinib.^7^ In patients with baseline extramedullary disease, the VGPR + CR rate difference was 18.8% (95% CI, 2.4 to 35.1) favoring zanubrutinib, consistent with the greater median reduction observed in lymphadenopathy (65.9% *v* 52.5%) and splenomegaly (20.0% *v* 15.0%) for zanubrutinib versus ibrutinib, respectively. VGPR + CR rates were 36.8% versus 22.2% in patients on zanubrutinib versus ibrutinib, respectively, with zero lines of prior therapy; 36.8% versus 25.7% with one to three lines of prior therapy; 28.6% versus 28.6% with greater than three lines of prior therapy. One CR was reported (cohort 2); the VGPR + CR rate was 30.8% and the major response rate was 65.4% in 26 patients with confirmed *MYD88*^WT^ WM.

Fewer progression-free survival (PFS; hazard ratio [HR], 0.63 [95% CI, 0.36 to 1.12]) and overall survival (OS; HR, 0.75 [95% CI, 0.36 to 1.59]) events were observed on zanubrutinib (cohort 1); median PFS or OS were not reached in the intent-to-treat population (Table 2; Figs 1B and 1C). In patients with *CXCR4*^MUT^ WM on ibrutinib, the median PFS was 39.8 months (Data Supplement, Figs 2 and 3). In cohort 2, 42-month event-free rates for PFS were lower than cohort 1; OS was comparable between cohorts (87.5% *v* 83.9%; Table 2; Data Supplement [Fig 4]).

### Long-Term Safety

Most common reasons for discontinuing treatment were AEs (cohort 1: nine zanubrutinib, 20 ibrutinib; cohort 2: six) and disease progression (cohort 1: 14 zanubrutinib, 13 ibrutinib; cohort 2: eight; Data Supplement [Fig 1]). Median treatment duration and relative dose intensities were similar between arms (cohort 1).

Any-grade AEs of diarrhea, muscle spasms, hypertension, atrial fibrillation/flutter, and pneumonia were more common with ibrutinib versus zanubrutinib; neutropenia was less common with ibrutinib versus zanubrutinib (cohort 1; Data Supplement, Tables 1 and 2). Incidences of AEs observed with zanubrutinib were similar between cohorts (Data Supplement, Table 3). More patients on ibrutinib experienced cardiovascular AEs, including one incidence of ventricular arrhythmia (Data Supplement, Table 4).

Except for neutropenia, prevalence of AEs of interest (Data Supplement, Table 5) were lower with zanubrutinib than ibrutinib at all time points (Fig 1D). Exposure-adjusted incidences of atrial fibrillation/flutter, hypertension, and diarrhea were significantly lower with zanubrutinib versus ibrutinib, respectively (descriptive *P* < .05; Data Supplement [Fig 5]). With zanubrutinib, the prevalence of neutropenia and infection decreased over time. By >36 months of treatment, the prevalence of infection was lower in patients receiving zanubrutinib than ibrutinib; the prevalence of neutropenia was similar between arms (Fig 1D).

More patients on ibrutinib than zanubrutinib required dose reductions because of AEs (cohort 1; Data Supplement [Table 3]). Treatment-emergent AEs led to discontinuation in 20 (20.4%) patients on ibrutinib versus nine (8.9%) on zanubrutinib. Most common AEs leading to discontinuation with ibrutinib were cardiac disorders and infections and infestations, with zanubrutinib as second malignancy (Data Supplement, Table 3). Higher risk of treatment discontinuation because of AEs (*P* < .05) and initiation of next treatment (*P* = .0977) was observed for ibrutinib versus zanubrutinib (Data Supplement, Figs 6 and 7).

Eight AE-related deaths occurred in cohort 1 (five ibrutinib; three zanubrutinib); three AE-related deaths occurred in cohort 2 (Data Supplement, Table 3).

## DISCUSSION

In ASPEN, zanubrutinib demonstrated meaningful efficacy by consistently exhibiting high-quality responses and favorable safety across 2 years of additional follow-up. High VGPR + CR rates observed with zanubrutinib across mutational groups also reflect a clinical benefit because achieving immunoglobulin M (IgM) reduction of >90% is associated with less IgM-related morbidity.

In other studies, no patients with *MYD88*^WT^ WM achieved a major response with ibrutinib or a VGPR/CR with acalabrutinib.^8,9^ In the ASPEN study, 31% of patients with *MYD88*^WT^ WM achieved a VGPR/CR with zanubrutinib, including one CR, after 44-month follow-up. Furthermore, PFS and OS in patients with *MYD88*^WT^ WM in our study were compared favorably with those receiving ibrutinib ± rituximab treatment in other studies, although all were limited by small sample size, and cross trial comparison was not possible.^10,11^ Our findings support zanubrutinib as the preferred treatment for patients with *MYD88*^WT^ WM.

Zanubrutinib exhibited fewer side effects associated with off-target binding, especially cardiovascular toxicities. With zanubrutinib, no cases of ventricular arrhythmia were observed; neutropenia occurred early and was neither treatment-limiting nor associated with a higher infection rate. Zanubrutinib was associated with longer treatment duration and lower risk of dose reduction or discontinuation because of AEs.^12^ Patients previously intolerant to ibrutinib or acalabrutinib did not experience a recurrence of treatment-related AEs with zanubrutinib.^12^

Study limitations include an open-label design, unknown *CXCR4* mutational status, and more patients with *CXCR4* mutations randomly assigned to zanubrutinib versus ibrutinib (cohort 1), all of which may have influenced the VGPR + CR rates observed. VGPR + CR rate was chosen as the primary end point for this study because of the prolonged responses and infrequent PFS/OS events expected and because response rates and depth of response are associated with PFS and time to next treatment in patients with WM.^13-15^ Although potential false negatives may have occurred because of assay sensitivity or lower bone marrow disease involvement in patients with *MYD88*^WT^ WM, the assay was sufficient for detection congruent with expected mutation rates.^16^ Potential associations between *CXCR4* nonsense versus frameshift mutations and treatment outcomes were evaluated (manuscript in preparation).

Extended follow-up results confirm improved long-term safety and tolerability of zanubrutinib compared with ibrutinib and support deeper, earlier, and more durable responses in patients with WM regardless of previous treatment or *CXCR4* and *MYD88* mutational statuses.

## Data Availability

The redacted study Protocol is provided online only with this article. All authors had access to the original data for the analyses described here. On request and subject to certain criteria, conditions, and exceptions, BeiGene will provide access to individual deidentified participant data from BeiGene-sponsored global interventional clinical studies conducted for medicines (1) for indications that have been approved or (2) in programs that have been terminated. Data requests may be submitted to DataDisclosure@beigene.com.
